# Chronic osteomyelitis risk is associated with *NLRP3* gene rs10754558 polymorphism in a Chinese Han Population

**DOI:** 10.1186/s12920-024-01799-6

**Published:** 2024-01-29

**Authors:** Yu-dun Qu, Nan Jiang, Jia-xuan Li, Wei Zhang, Chang-liang Xia, Shuan-ji Ou, Yang Yang, Yun-fei Ma, Yong Qi, Chang-peng Xu

**Affiliations:** 1 Department of Orthopaedics, Guangdong Second Provincial General Hospital, 466 Xingang Road, Haizhu District, 510317 Guangzhou, China; 2 Division of Orthopaedics and Traumatology, Department of Orthopaedics, Nanfang Hospital, Southern Medical University, 510515 Guangzhou, China

**Keywords:** Single nucleotide polymorphisms, Chronic osteomyelitis, rs10754558, rs7525979, Case-control study

## Abstract

**Background:**

Single nucleotide polymorphisms (SNPs) in the nucleotide-binding domain leucine-rich repeat protein-3 (*NLRP3*) gene are reported to be linked to many inflammatory disorders. However, uncertainty persists over the associations between these SNPs and susceptibilities to chronic osteomyelitis (COM). This study aimed to investigate potential relationships between *NLRP3* gene SNPs and the risks of developing COM in a Chinese Han cohort.

**Methods:**

The four tag SNPs of the *NLRP3* gene were genotyped in a total of 428 COM patients and 368 healthy controlsusing the SNapShot technique. The genotype distribution, mutant allele frequency, and the four genetic models (dominant, recessive, homozygous, and heterozygous) of the four SNPs were compared between the two groups.

**Results:**

A significant association was found between rs10754558 polymorphism and the probability of COM occurence by the heterozygous model (*P* = 0.037, odds ratio [OR] = 1.541, 95% confidence interval [CI] = 1.025–2.319), indicating that rs10754558 may be associated with a higher risk of developing COM.In addition, possible relationship was found between rs7525979 polymorphism and the risk of COM development by the outcomes of homozygous (*P* = 0.073, OR = 0.453, 95% CI = 0.187–1.097) and recessive (*P* = 0.093, OR = 0.478, 95% CI = 0.198–1.151) models, though no statistical differences were obtained.

**Conclusions:**

Outcomes of the present study showed, for the first time, that rs10754558 polymorphism of the *NLRP3* gene may increase the risk of COM development in this Chinese Han population, with genotype CG as a risk factor. Nonetheless, this conclusion requires verification from further studies with a larger sample size.

**Supplementary Information:**

The online version contains supplementary material available at 10.1186/s12920-024-01799-6.

## Background

Chronic osteomyelitis (COM) is a frequent and challenging complication of trauma, affecting up to one third of patients during recovery from severe limb injury or open fracture [1, 2]. In addition, hematogenous seeding and soft tissue infections, especially in patients with diabetes and pressure ulcers, can also lead to osteomyelitis (OM) [3]. *Staphylococcus aureus* remains to be the primary pathogen of COM [4, 5]. Owing to insufficient blood supply to the infected bone, patients with COM often require both surgical debridement and antibiotic treatment [6, 7]. COM has been shown to substantially increase the long-term mortality risk [8]. Approximately 30% of the acute OM cases progress into chronic phase [9], causing anincreased risk of mortality, perpetuating disability, and worsened quality of life [8, 10, 11]. Previous epidemiological studies have reported that COM elevates the risk of coronary heart disease [12], stroke [13], diabetes mellitus [14], renal disease [15], and even depression [10]. To comprehensively tackle COM-related issues, substantial efforts should be devoted to investigate the pathophysiology of the condition as well as to conduct clinical studies. The pathogenesis of OM is related to both environmental factors and genetic factors, and contemporary evidence suggests that genetic susceptibility may also play a crucial role in this process [16, 17]. With the rapid development and application of sequencing and genetic association analysis, genetic variants that may cause OM have been extensively explored.

Numerous genetic studies examining nucleotide-binding domain leucine-rich repeat protein-3 (*NLRP3*) polymorphisms have demonstrated the involvement of this gene in a range of inflammatory conditions, including Parkinson’s and Alzheimer’s diseases [18], type 2 diabetes, atherosclerosis [19], inflammatory bowel disease [20], gout, and recurrent fever [21]. Genetic differences in the *NLRP3* have been hypothesized to have a substantial role in determining the severity of inflammatory responses, thereby predisposing susceptibility to disease [22]. However, the association between *NLRP3* and COM, as well as the underlying mechanisms remain unclear. Previous studies had reported that several single nucleotide polymorphisms (SNPs), such as *TaqI* (rs731236) and *FokI* (rs2228570) in the *vitamin D receptor* (*VDR*) gene [23], rs689466 in the *cyclooxygenase-2* (*COX-2*) gene [24], the Alu insertion/deletion (rs4646972) in the *tissue plasminogen activator *(*tPA*) gene [25], and rs1799750 in the *matrix metalloprotease 1* (*MMP-1*) gene [26], may be associated with the risk of developing COM. In the HaploReg v4.2-Broad Institute (HaploReg v4.2 (broadinstitute.org)), we found that rs10754558, rs7525979, rs35829419, and rs4612666 were all involved in inflammatory responses and all played key roles in the mouse OM model (Supplementary Table S1).

To better understand potential role of genetic factor in the pathogenesis of COM, this study examined potential links between *NLRP3 * SNPs and susceptibilities to COM in a Chinese Han population. 

## Materials and methods

### Study design and patient enrollment 

This investigation was designed as a case-control study and conducted in a Chinese Han population. Patients undergoing treatment for COM between 2016 and 2019 at the Nanfang Hospital, Southern Medical University, a tertiary healthcare facility in Southern China, were screened for enrollment. COM was defined by symptoms persisting for at least 10 weeks and/or radiological appearances suggestive signs of bone infection. In addition, at least one of the following operative findings was required to be present for inclusion: (i) two or more positive culture sterile site specimens with an indistinguishable organism; (ii) histology suggestive of COM (a mean of > 5 neutrophils per high power field, averaged over at least 5 fields); or (iii) sinus abscess or purulence, present during surgery. These criteria align with established methods of confirming the diagnosis of COM. Healthy controls were individuals without abnormalities or a history of any disorders, as determined by thorough examinations in the physical examination center. Informed consent forms were completed by all the participants or their guardians, and the medical ethics committee of the Southern Medical University Nanfang Hospital approved the study (approval no. NFEC-2019-087).

### SNP selection and genotyping

Data regarding *NLRP3* gene expression in osteoblasts were obtained through gene sequencing (BioProject number: PRJNA1001643). The model included a normal osteogenic differentiation group (tumor necrosis factor [*TNF-α*] = 0 ng/mL) and an inflammasome group (*TNF-α* = 5 ng/mL). The expression of the *NLRP3* gene was then compared between the samples belonging to these two groups. We analyzed four selected SNPs (rs35829419, rs10754558, rs7525979, and rs4612666) in the *NLRP3* inflammasome gene. Previous studies on inflammasome gene polymorphisms related to infection were referred. The expressions of these four SNPs in tissues were verified in the Genotype-Tissue Expression (GTEx) database. The GTEx project is an ongoing effort to build a comprehensive public resource to study tissue-specific gene expression and regulation. It provides open access to information, including gene expression, quantitative trait loci data, and histology images.

Peripheral blood samples (2 mL) were collected from each participant in tubes containing ethylenediaminetetraacetic acid. Polymerase chain reaction (PCR) was performed in a total reaction volume of 15 µL, which contained 1.0 µL of template DNA, 1.5 µL of 10× PCR buffer, 1.5 µL of 25 mmol/L MgCl_2_, 0.3 µL of 10 mmol/L dNTP mix, 0.15 µL of 10 µmol/mL primers, and 0.3 µL of 5 µ/µL Taq DNA polymerase (Fermentas, Waltham, MA, USA). Table 1 lists the forward, reverse, and extension primers used [23]. Reactions were performed using a PCR machine (model no. EDC-810, Dongsheng Co., Ltd., Beijing, China) with the following settings: initial denaturation at 94 °C for 3 min, followed by 35 cycles of denaturation at 94 °C for 15 s, annealing at 55 °C for 15 s, extension at 72 °C for 30 s, and a final extension step at 72 °C for 3 min. PCR products were purified in the following mixture: 3.0 µL of PCR products, 0.2 µL of 20 µ/µL Exonuclease I (ExoI; Fermentas), 0.8 µL of 1 µ/µL FastAP thermosensitive alkaline phosphatase (Fermentas), 0.7 µL of ExoI buffer, and 2.3 µL of ddH_2_O. Reactions were conducted at 37 °C for 15 min and 80 °C for 15 min.

**Table 1:** Forward, reverse, and extension primers of the 4 tag SNPs for PCR

SNPs	Forward and reverse primers (5’-3’)	Extension primers (5’-3’)
rs10754558	F: 5’-GTGGAGTGTCGGAGAAGAGA-3’ R: 5’-GCTAATTACATGAGGTCACCAAGA-3’	5’-CTGACTGACTGACTGACTGACTGACTGCAATGACAGCATCGGGTGTTGTT-3’
rs7525979	F: 5’-CCATCGGCAAGACCAAGAC-3’ R: 5’-CCAGGCTCCTCTGTGTCA-3’	5’-CTGACTGACTGACTGACTGACTGACTGACTGACTGACTGACTGACTGTTCTGAGCCTGTGCACAC-3’
rs35829419	F: 5’-CTGTCATCGGGTGGAGTCA-3’ R: 5’-GCGAGGAAGCAGGAGGAA-3’	5’-CTGACTGACTGACTGACTGAGAGGAGCTTGGGAGGACACACT-3’
rs4612666	F: 5’-GGTTGCACAACAATGTGAAGT-3’ R: 5’-ATAACAAGTAAGCATTCTCCAAGC-3’	5’-TTCTCCAAGCTCCCACCAATACTAC-3’

SNP: Single Nucleotide Polymorphism; PCR: Polymerase Chain Reaction; F: Forward; R: Reverse

**Fig. 1:**
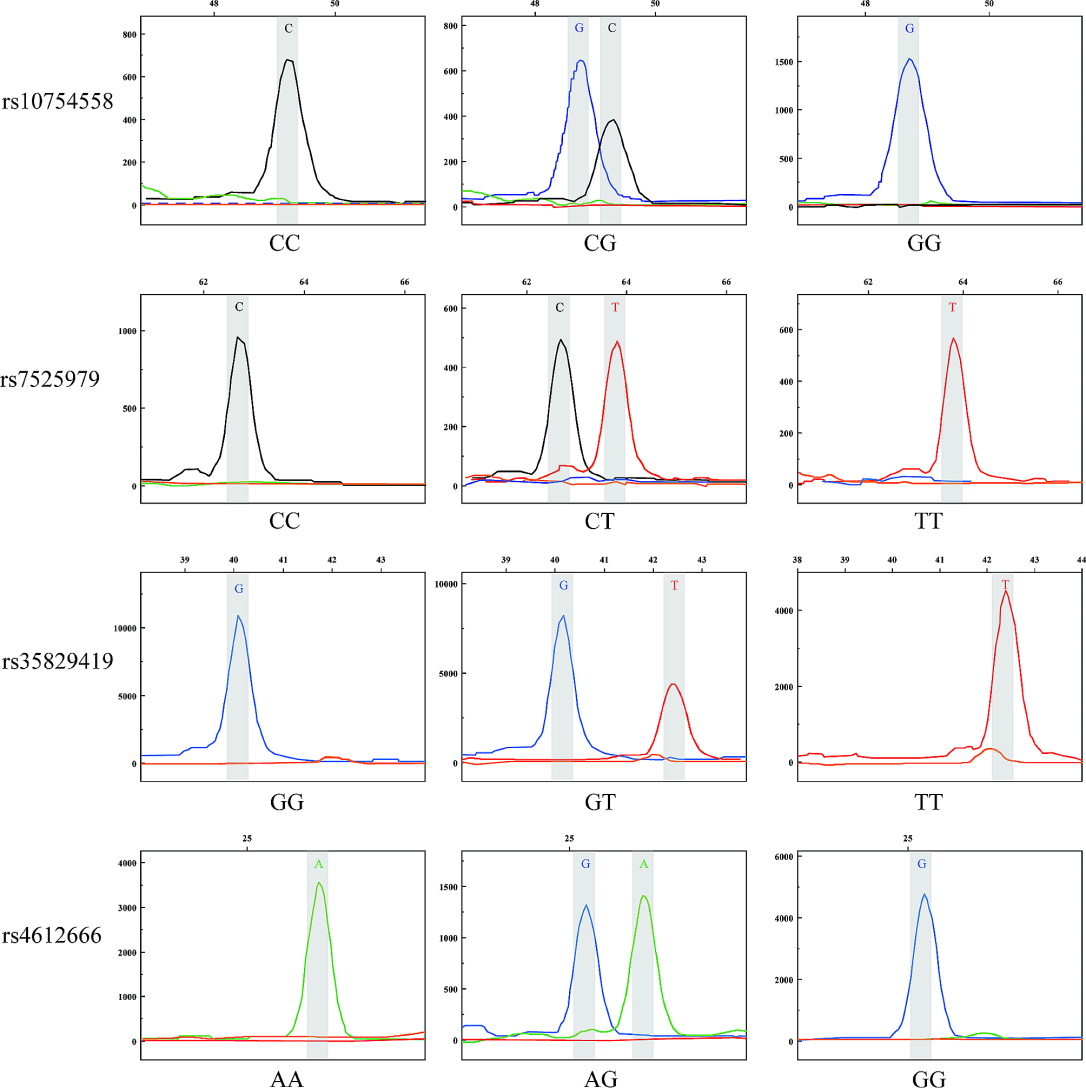
SNaPshot genotyping. The rs10754558, rs7525979, rs35829419, and rs4612666 single nucleotide polymorphisms of the nucleotide-binding domain and leucine-rich repeat protein-3 gene were analyzed using the SNaPshot technique (Supplementary Table S2)

Using the Multiplex SNaPshot technique, the four SNPs of the *NLRP3* gene were genotyped. SNaPshot extension reactions were performed following the instructions of the ABI SNaPshot Multiplex PCR Kit (Applied Biosystems, Waltham, MA, USA) with slight revisions. The total reaction volume (6.0 µL) included 2.0 µL of purified PCR products, 1.0 µL of SNaPshot Multiplex Ready Reaction Mix, 0.2 µL of 10 µmol/mL pooled extension primer, and 2.8 µL of ddH_2_O. Extension reactions were performed as follows: pre-denaturation at 96 °C for 1 min, followed by 30 cycles of denaturation at 96 °C for 10 s, annealing at 52 °C for 5 s, and extension at 60 °C for 30 s. The product of this reaction (1.0 µL) was mixed with 9.0 µL of formamide and denatured at 95 °C for 3 min. The fluorescently labeled fragments were separated by capillary electrophoresis on an ABI PRISM 3730 XL Genetic Analyzer (Applied Biosystems). The results of the SNaPshot genotyping method are shown in Fig. 1.

### Comparative analysis of *NLRP3* gene polymorphisms and clinical indicators

Genotype distribution, mutant allele frequency, and the four genetic models (dominant, recessive, homozygous, and heterozygous) of the four *NLRP3* SNPs were compared between the COM patients and healthy controls. Age, sex ratio, culture-positive rate, polymicrobial infection rate, and preoperative serum levels of two inflammatory biomarkers, interleukin (*IL*)-6 and *TNF-α*, were compared between the four genotypes of *NLRP3* gene polymorphisms among the patient group. Serum levels of *IL-6* and *TNF-α* were detected using electrochemiluminescence immunoassay (Roche cobas e601, Basel, Switzerland). The upper limit of normal values, provided by the Medical Clinical Laboratory of Nanfang Hospital, were 7.0 and 8.1 pg/mL for IL-6 and TNF-α, respectively.

### Statistical analysis

The Kolmogorov–Smirnov test assessed data normality. Normally distributed continuous variables are reported as the mean ± standard deviation, and a one-way analysis of variance or the student’s t-test was used to compare between the two or among over two groups. Continuous variables with a non-normal distribution were reported as the median withinterquartile range (IQR), and the Kruskal–Wallis or Mann–Whitney U tests were used for group comparisons. Dichotomous variables with percentage-based data were compared using the Chi-square or the Fisher’s exact test.

Analysis of the linkage disequilibrium at different loci of *NLRP3* genotypes was performed. The genotype distributions of the healthy controls were examined using the Chi-square test to determine whether Hardy–Weinberg equilibrium (HWE) were sustained. The Chi-square test or Fisher’s exact test was used to examine the genotype distributions and mutant allele frequencies between the two groups. Sex, age, and genotype distribution were covariates. Binary logistic regression analysis was performed to examine the potential relationships between the four gene polymorphisms and the likelihood of developing COM using four genetic models with matching odds ratios (ORs) and 95% confidence intervals (CI). Statistical analyses were performed using R software (Version 4.2.3, LD-heatmap package) and SPSS version 25.0 (IBM Corp., Armonk, NY, USA). Statistical significance was established as a *P*-value < 0.05.

## Results

### Clinical characteristics of the participants

**Fig. 2:**
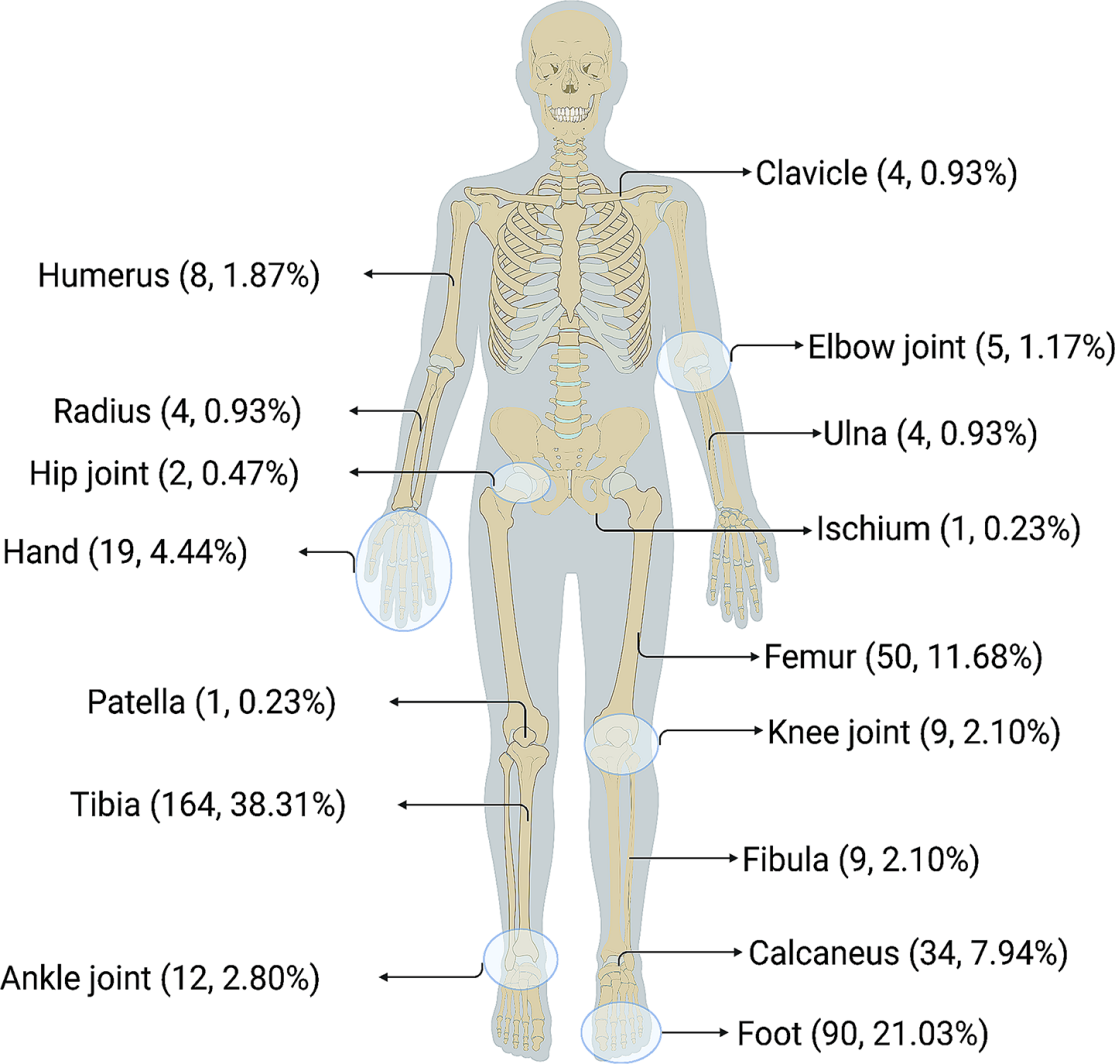
Distribution map of infection sites of the COM patients. The number of patients involved is given at each site, along with the percentage of the total number of 428 patients

This study included 368 healthy controls (268 men and 100 women) and 428 COM patients (337 men and 91 women). There was no statistically significant difference in the sex ratio between the two groups (3.70 vs. 2.68, χ^2^ = 3.792, *P* = 0.051). The median ages of the two groups were not significantly different (patient group: 47 years, IQR, 33.00–59.00; control group: 46 years, IQR, 37.00–52.00; *Z* = 0.114, *P* = 0.662). Figure 2 shows clinical characteristics of COM of this Chinese cohort. The most prevalent type of COM was post-traumatic OM (PTOM)(58.35%), which often occurred after open injury (61.96%). The most typical infection location was the tibia (38.31%), followed by the foot (21.03%) and the femur (11.68%). Of all the intraoperative specimen cultures, 27.46% were tested positive, with monomicrobial infection accounting for 71.67%. The most commonly detected pathogen was *Staphylococcus aureus* (34.17%).

### Expression of *NLRP3* in different states and linkage disequilibrium analysis

**Fig. 3:**
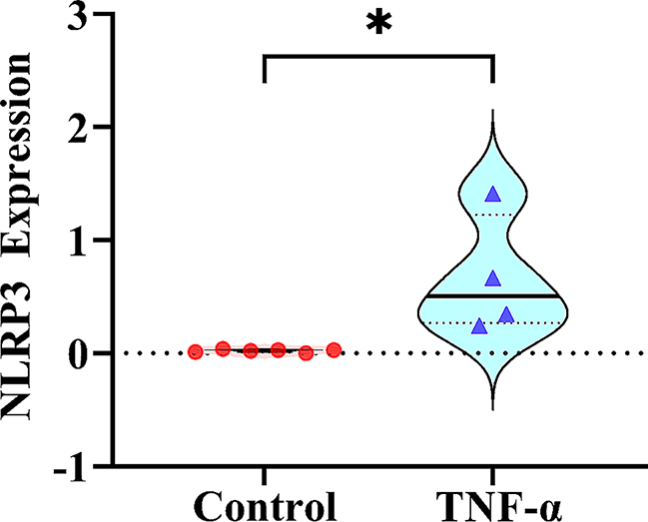
Expression of the *NLRP3* gene in osteoblasts. *NLRP3* gene expression was analyzed in osteoblasts cultured under normal conditions (control) and those cultured in the presence of TNF-α (inflammatory microenvironment). *NLRP3*, nucleotide-binding domain and leucine-rich repeat protein-3; TNF-α, tumor necrosis factor-α, *P = 0.015

**Fig. 4:**
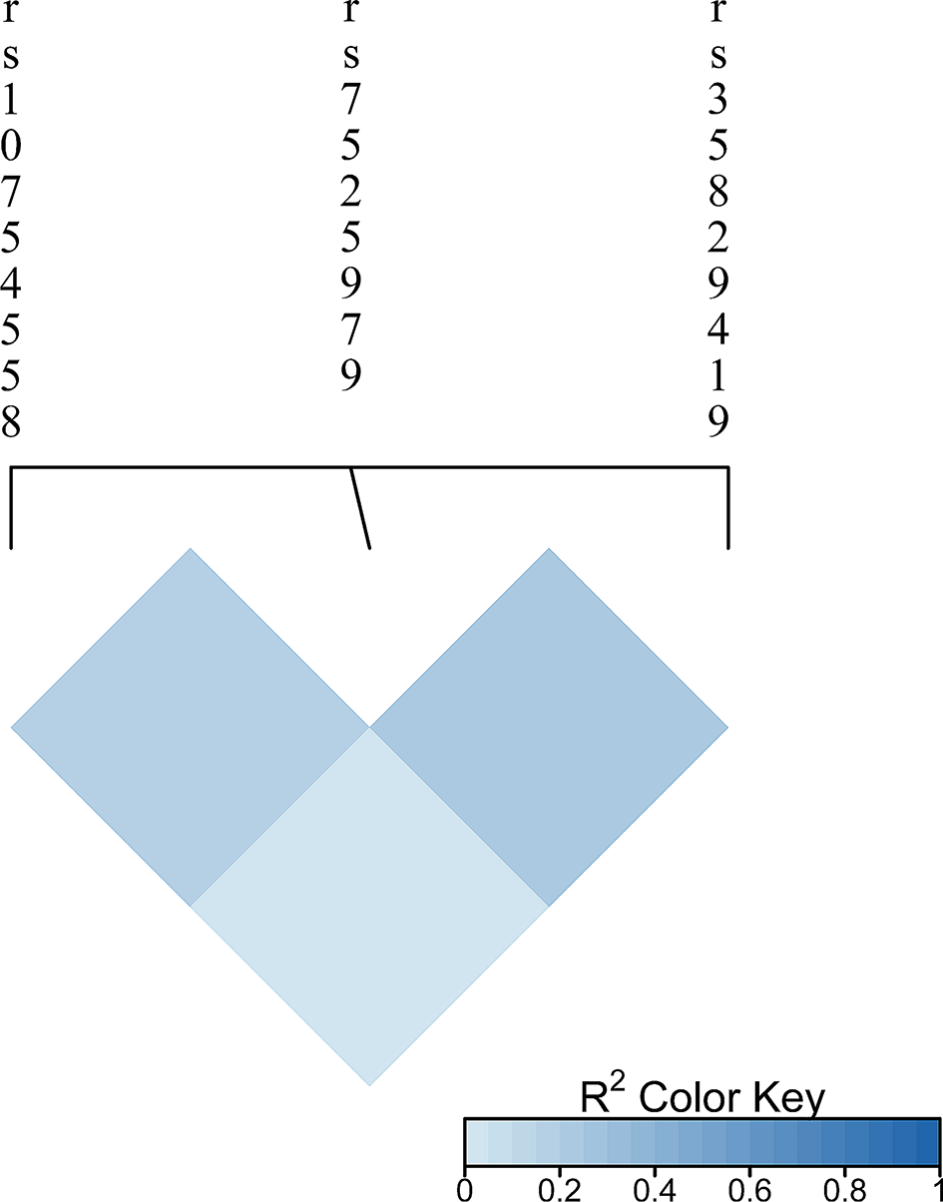
Linkage disequilibrium analysis. The r^2^ hot graph for the single nucleotide polymorphisms of the nucleotide-binding domain and leucine-rich repeat protein-3 gene: rs10754558, rs7525979, and rs35829419

 The expression of the *NLRP3* gene under inflammatory microenvironment was significantly higher than that under normal microenvironment (*P* = 0.015) (Fig. 3). However, according to the GTEx database, the expression of *NLRP3* gene variants (rs10754558, rs7525979, rs35829419, and rs4612666) in the whole-blood and the musculoskeletal tissues did not show significant differences. The r^2^ values of the *NLRP3* gene SNPs were calculated using *R* software (Version 4.2.3). The results of the linkage disequilibrium analysis were shown in the r^2^ hot graph in Fig. 4. Deep-blue blocks represent high linkage disequilibrium (r^2^ = 0.8–1), and light-blue blocks represent low linkage disequilibrium (r^2^ = 0–0.2). There was no linkage disequilibrium among the three SNPs of *NLRP3* (rs10754558, rs7525979, and rs35829419).

### Frequency of the four *NLRP3* gene SNPs in COM patients and healthy controls

In the healthy controls, all the four genotyped *NLRP3* gene SNPs were in HWE: *P*_(HWE)_ for rs35829419 = 0.979, *P*_(HWE)_ for rs10754558 = 0.377, *P*_(HWE)_ for rs7525979 = 0.386, and *P*_(HWE)_ for rs4612666 = 0.607. As shown in Table 2, the heterozygous model of the rs10754558 SNP and susceptibility to COM were significantly associated (*P* = 0.037, OR = 1.541, 95% CI = 1.025–2.319), indicating that individuals with the CG genotype may be at a higher risk in developing COMin this Chinese Han population.

**Table 2:** Comparisons of genotype distribution, allele frequency, and genetic models of rs10754558, rs7525979, rs35829419 and rs4612666 between the COM patients and healthy controls

SNPs	Item	Allele or genotype	Patients	Controls	*P* values	OR (95% CI)
rs10754558	Genotype (n, %)	CC	139 (32.48)	135 (36.68)	0.053	NA
		CG	232 (54.21)	169 (45.92)		
		GG	57 (13.32)	64 (17.39)		
	Allele frequency	C vs. G	510/346	439/297	0.978	0.997 (0.816–1.219)
	Dominant model	CC + CG vs. GG	371/57	304/64	0.110	1.370 (0.930–2.019)
	Recessive model	CC vs. CG + GG	139/289	135/233	0.213	0.830 (0.619–1.113)
	Homozygous model	CC vs. GG	139/57	135/64	0.507	1.156 (0.753–1.774)
	Heterozygous model	CG vs. GG	232/57	169/64	**0.037**	**1.541 (1.025**–**2.319)**
rs7525979	Genotype (n, %)	TT	8 (1.87)	14 (3.84)	0.120	NA
		CT	106 (24.77)	102 (27.95)		
		CC	314 (73.36)	249 (68.22)		
	Allele frequency	T vs. C	122/734	130/600	0.054	0.767 (0.586–1.004)
	Dominant model	TT + CT vs. CC	114/314	116/249	0.112	0.779 (0.573–1.060)
	Recessive model	TT vs. CT + CC	8/420	14/351	0.093	0.478 (0.198–1.151)
	Homozygous model	TT vs. CC	8/314	14/249	0.073	0.453 (0.187–1.097)
	Heterozygous model	CT vs. CC	106/314	102/249	0.234	0.824 (0.599–1.133)
rs35829419	Genotype (n, %)	AA	0 (0.0)	0 (0.0)	NA	NA
		AC	3 (0.70)	1 (0.27)		
		CC	424 (99.30)	367 (99.73)		
	Allele frequency	A vs. C	3/851	1/735	0.724	2.591 (0.269–24.963)
	Dominant model	AA + AC vs. CC	3/424	1/367	0.724	2.597 (0.269– 25.071)
	Recessive model	AA vs. AC + CC	0/427	0/368	NA	NA
	Homozygous model	AA vs. CC	0/424	0/367	NA	NA
	Heterozygous model	AC vs. CC	3/424	1/367	0.724	2.597 (0.269–25.071)
rs4612666	Genotype (n, %)	CC	114 (26.64)	98 (26.63)	0.422	NA
		CT	224 (52.34)	179 (48.64)		
		TT	90 (21.03)	91 (24.73)		
	Allele frequency	C vs. T	452/404	375/361	0.461	1.077 (0.884–1.312)
	Dominant model	CC + CT vs. TT	338/90	277/91	0.214	1.234 (0.885–1.719)
	Recessive model	CC vs. GT + TT	114/314	98/270	0.999	1.000 (0.730–1.371)
	Homozygous model	CC vs. TT	114/90	98/91	0.423	1.176 (0.791–1.750)
	Heterozygous model	CT vs. TT	224/90	179/91	0.189	1.265 (0.890–1.798)

COM: Chronic osteomyelitis; OR: odds ratio, CI: confidence interval, NA: not available

Although no significant association was identified between rs7525979 and the risk of developing COM, results of the recessive (*P* = 0.093) and homozygous (*P* = 0.073) models suggested that this SNP site may be linked to a decreased risk of COM development, with genotype TT as a possibly protective factor. However, this results need to be further tested. No significant correlations were found between rs35829419 or rs4612666 and the risks of COM development among these participants (Table 2).

### *IL-6* and *TNF-α* levels among different genotypes of rs10754558 and rs7525979 in the COM patients 

**Fig. 5:**
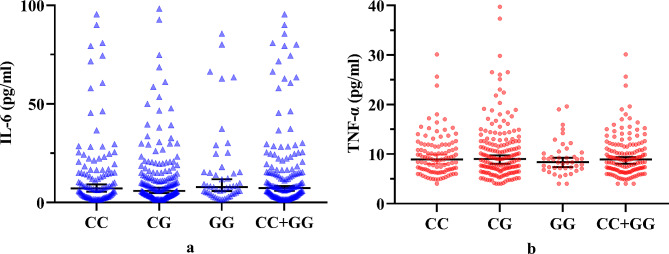
Cytokine levels in patients with chronic osteomyelitis. Serum levels of **a** IL-6 and **b** TNF-α were compared among different genotypes of the single nucleotide polymorphism rs10754558. IL-6, interleukin-6; TNF-α, tumor necrosis factor-α

**Fig. 6:**
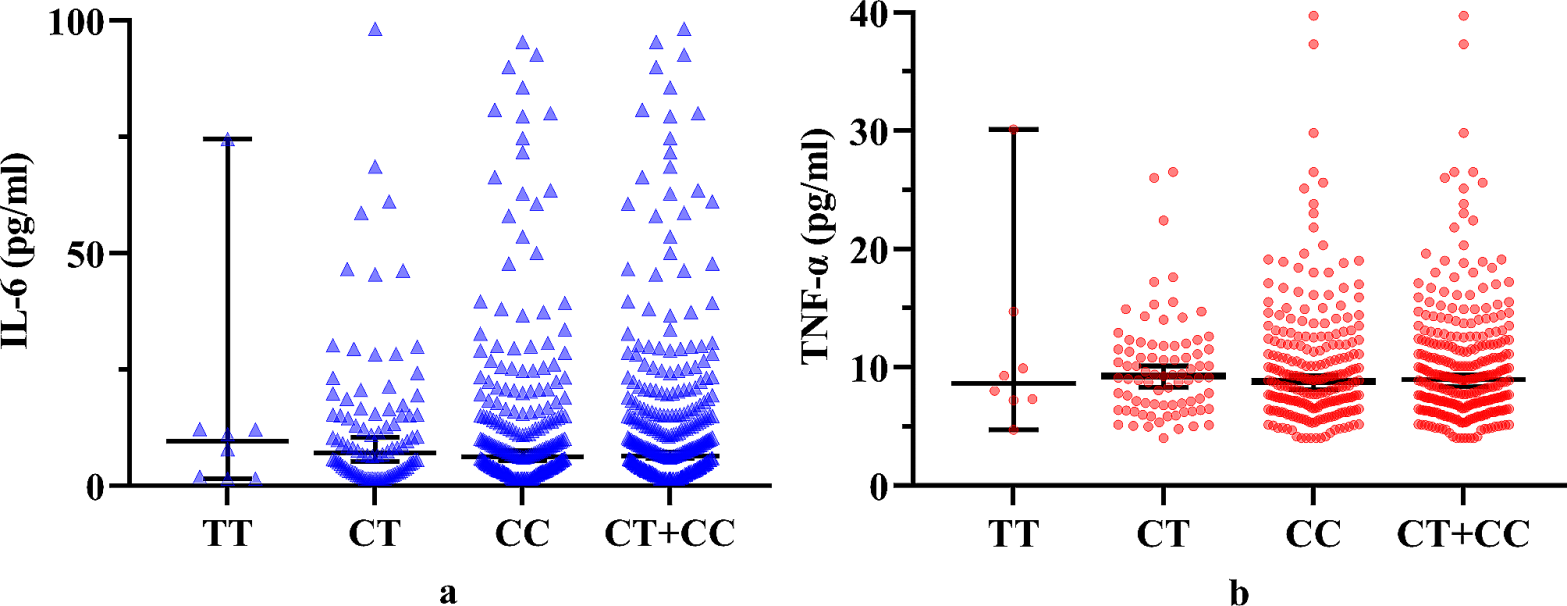
Cytokine levels in patients with chronic osteomyelitis. Serum levels of **a** IL-6 and **b** TNF-α were compared among different genotypes of the single nucleotide polymorphism rs7525979. IL-6, interleukin-6; TNF-α, tumor necrosis factor-α

Serum levels of *IL-6* and *TNF-α* in the COM patients showed no significant differences among different genotypes of rs10754558 (*P* = 0.213 and 0.662, respectively; Table 3). Additionally, multiple comparisons showed that no significant differences were found regarding serological *IL-6* or *TNF-α* levels between CG and GG (*P* = 0.180), CG and CC (*P* = 0.158), or CG and CC + GG (*P* = 0.072) genotype groups (Fig. 5). Moreover, serum levels of *IL-6* and *TNF-α* did not differ significantlyamong different genotypes of rs7525979 (*P* = 0.900 and 0.843, respectively; Table 3) (Fig. 6).

**Table 3:** Comparisons of clinical features among different genotypes of the *NLRP3* gene polymorphisms among the included COM patients

Items	rs10754558				rs7525979				rs35829419				rs4612666			
	CC	CG	GG	*P* value	TT	CT	CC	*P* value	AA	AC	CC	P value	CC	CT	TT	*P* value
Age Median (IQR)	46 (30.5, 58)	48 35.5, 59)	48 (33, 60)	0.747	52.5 (32.5, 70)	43 (31, 59)	48 (34.5, 59)	0.583	NA	59 (27, 59)	47 (33, 59)	0.616	48.5 (38.5, 59.25)	45 (32, 59)	48 (32.5, 61.75)	0.193
Sex ratio (M/F)	4.65	2.95	2.94	0.210	7.00	4.15	3.12	0.455	NA	NA	3.33	0.344	2.69	3.29	5.13	0.176
PTOM proportion (%)(PTOM/COM)	46.76	56.47	54.39	0.593	37.50	75.47	72.29	0.575	NA	66.67	72.64	0.647	66.67	75.45	72.22	0.789
Positive rate of culture (%)	40.30	32.80	32.20	0.553	37.50	42.10	32.80	0.302	NA	66.70	35.10	0.545	30.40	33.60	45.10	0.148
Polymicrobial infection (%)	16.50	15.10	13.80	0.645	25.00	18.70	14.00	0.580	NA	33.30	15.30	0.724	17.40	14.30	15.40	0.454
IL-6 (pg/ml) Median (IQR)	7.33 (2.87,16.57)	5.87 (3.18, 12.70)	7.83 (4,58, 16.12)	0.213	9.53 (1.65, 12.18)	7.10 (2.99, 16.20)	6.36 (3.20, 14.63)	0.900	NA	9.31 (6.01, 9.31)	6.54 (3.17, 14.79)	0.250	6.07 (3.34, 12.37)	6.64 (3.26, 15.08)	6.17 (2.50, 16.76)	0.939
TNF-α (pg/ml) Median (IQR)	8.92 (6.75, 11.88)	9.08 (6.87, 12.33)	8.34 (7.15, 10.30)	0.662	8.66 (7.25, 13.51)	9.30 (6.79, 11.83)	8.81 (6.84, 11.90)	0.843	NA	7.88 (6.15, 7.88)	8.98 (6.87, 11.90)	0.978	8.34 (6.81, 12.80)	9.04 (7.07, 11.65)	8.84 (6.39, 12.30)	0.972

COM: chronic osteomyelitis; PTOM: post-traumatic osteomyelitis; IQR: interquartile range; M/F: male/female; IL-6: interleukin-6; TNF-α: tumor necrosis factor-α

## Discussion

DOur study results showed that, in this ChineseHan cohort, *NLRP3* SNP rs1074558 may increase the risk of COM development, with CG genotype in a higher risk to develop COM. Despite the current study being unable to obtain statistical evidence supporting this association between the COM patients and healthy controls, the results of the recessive and homozygous models suggested that rs7525979 polymorphism may hinder COM development. Nonetheless, this conclusion requires verification from further studies with a larger sample size.

COM, which often develops following trauma and orthopaedic surgery, still poses great challenges tophysicians. The frequency of infection in the long bones after open fractures varies between 4% and 64%, with infection recurrence rates ranged between 20% and 30% [27]. Among the 428 COM patientsincluded in our study, PTOMaccounted for 58.35%, with infection following open injury occupying 61.96% and the positive rate of culture as27.46%. The physical and psychological well-being of patients is severely affected by the protracted course of the disease and inflammatory bone disintegration. Huang et al. [28] found that COM significantly increased the risk of death in the older population. Along with the aforementioned comorbidities, patients with COM also suffer fromhigher rates of deep vein thrombosis [15], erectile dysfunction [29], dementia [30], and end-stage renal disease [31], as well as mental health disorders, including depression [10]. In addition, while rare, there is a risk of malignant change in patients with COM [32].Consequently, COM is accompanied by an enormous healthcare and economic burdens [33].

Previous studies have found that *IL-1β* secretion and mRNA expression levels of key inflammasome components, namely apoptosis-associated speck-like protein containing a *CARD* and Caspase-1, are abnormally increased in patients with chronic recurrent multifocal osteomyelitis [34, 35]. The release of *IL-1β* can lead to autocrine stimulation and production of other cytokines, such as *IL-6*. In the present study, we did not detect the serological level of *IL-1β*. Thus, the potential influences of such SNPs on serological levels of IL-1β cannot be assessed. Instead, we evaluated the impact of genotype by comparing readily available serum inflammatory markers, *IL-6* and *TNF-α*.

Studies have shown that genetic variables, with SNP as a key component, may play a role in the etiology of infection. According to several clinical studies, the *VDR* [23], *tPA* [25], *COX-2* [24], and *MMP-1* [26] genes, and the most frequently reported members of the *IL* family, have been implicated in the associations between SNPs and COM risk across various ethnicities. Previous investigations reported that *IL*-*1* (rs1800587) [36, 37], *IL*-*4* (rs2243250, rs2243248) [37], and *IL*-*6* (rs1800795) [37] gene polymorphisms are positively correlated with the development of COM and raise the risk of developing OM. And we found that these SNPs pathways are associated with inflammation. As a crucial component of the inflammatory system, the *NLRP3* inflammasome may also be linked to the incidence and progression of COM [38]. More importantly, our team investigated a possible association between *NLRP3* gene polymorphisms and the risks of developing PTOM in a Chinese population. The results suggested that *NLRP3* SNPs, rs10754558 *(P =* 0.047) and rs7525979 *(P* = 0.048), were significantly different between patients and healthy controls. However, this study only discussed the susceptibility to PTOM and *NLRP3* gene expressions, and could not clarify whether this gene susceptibility was related to COM. At the same time, we summarized the SNPs that are associated with COM, as well as the mechanistic pathways involved and the action (cis or trans) and location of SNPs (Table 4). Therefore, we conducted a more detailed study [39].

**Table 4:** Previously reported SNP sites associating with OM development

PMID	Gene	SNPs	Chr: Pos	Action of SNPs	Pathway*	OM or OM type
35957979	*VDR*	rs7975232	12:48238837	Cis-eQTL	Generic Transcription Pathway	COM
		rs1544410	12:48239835	Cis-eQTL	Generic Transcription Pathway	
34628386	*IL-1β* *PON1*	rs1143634	2:113590390	Cis-eQTL	Interleukin-4 and Interleukin-13 signaling	OM
		rs705379	7:94953895	Not found	Atorvastatin ADME	
32542640	*CRP*	rs11265260	1:159700039	Not found	Classical antibody-mediated complement activation	DFO
		rs1800947	1:159683438	Not found	Classical antibody-mediated complement activation	
		rs3093059	1:159685136	Not found	Classical antibody-mediated complement activation	
		rs1130864	1:159683091	Not found	Classical antibody-mediated complement activation	
32454789	*IL-1β* *IL-6* *IL-1RN*	rs16944	2:113594867	Cis-eQTL	Signaling by Interleukins	PTOM
		rs1143627	2:113594387	Cis-eQTL	Cell recruitment (pro-inflammatory response)	
		rs1800796	7:22766246	Cis-eQTL	Not found	
		rs4251961	2:113874467	Cis-eQTL	Interleukin-1 family signaling	
31746120	*IFN-γ*	rs2430561	12:68552522	Cis-eQTL	Not found	PTOM
31647805	*CTSG*	rs45567233	14:25043671	Cis-eQTL	Cell recruitment (pro-inflammatory response)	OM
30949508	*IL-1β*	rs16944	2:113594867	Cis-eQTL	Interleukin-1 family signaling	COM
30259788	*TNF-α*	rs1799964	6:31542308	Cis-eQTL	Cytokine Signaling in Immune system	COM
29049163	*MMP-1*	rs1799750	11:102670496	Cis-eQTL	Cytokine Signaling in Immune system	OM
		rs1144393	11:102669409	Cis-eQTL	Cytokine Signaling in Immune system	
27329975	*VDR*	rs731236	12:48238757	Cis-eQTL	Vitamin D (calciferol) metabolism	COM
		rs2228570	12:48272895	Cis-eQTL	Generic Transcription Pathway	
27323068	*IL-1β* *TLR-2*	rs16944	2:113594867	Cis-eQTL	Vitamin D (calciferol) metabolism	HO
		rs3804099	4:154624656	Cis-eQTL	Neutrophil degranulation	
26681045	*IL-4* *IL-10*	rs2070874	5:132009710	Cis-eQTL	Cytokine Signaling in Immune system	HO
		rs1800871	1:206946634	Cis-eQTL	CD163 mediating an anti-inflammatory response	
28682162	*COX-2*	rs689466	1:186650751	Cis-eQTL	Interleukin-10 signaling	PTOM
23570848	*tPA*	rs4646972	Not found	Not found	Not found	OM
19821768	*MMP-1*	rs1799750	11:102670496	Cis-eQTL	Regulation of Insulin-like Growth Factor (IGF) transport and uptake by Insulin-like Growth Factor Binding Proteins (IGFBPs)	OM
18971305	*IL-1α* *IL-4* *IL-6*	rs1800587	2:113542960	Cis-eQTL	Cell recruitment (pro-inflammatory response)	COM
		rs2243248	5:132008644	Cis-eQTL	Interleukin-4 and Interleukin-13 signaling	
		rs2243250	5:132009154	Cis-eQTL	Cytokine Signaling in Immune system	
		rs1800795	7:22766645	Not found	Not found	
17438390	*BAX*	rs4645878	19:49457938	Cis-eQTL	Apoptosis	OM
16487238	*TLR4*	rs4986790	9:120475302	Cis-eQTL	MyD88 dependent cascade initiated on endosome	HO
The present study	*NLRP3*	rs10754558	1:247612036	Cis-eQTL	Nucleotide-binding domain, leucine rich repeat containing receptor (NLR) signaling pathways	COM
		rs7525979	1:247587408	Cis-eQTL	Nucleotide-binding domain, leucine rich repeat containing receptor (NLR) signaling pathways	

OM: osteomyelitis; COM: chronic osteomyelitis; DFO: diabetic foot osteomyelitis; PTOM: post-traumatic osteomyelitis; HO: hematogenous osteomyelitis. *VDR*: vitamin D receptor; *IL*: interleukin; *PON1*: paraoxonase-1; *CRP*: c-reactive protein; *IFN*: interferon; *CTSG*: cathepsin G; *TNF-α*: tumor necrosis factor-α; *MMP-1*: matrix metalloproteinase-1; *TLR*: toll-like receptor; *COX-2*: cyclooxygenase-2; *tPA*: tissue plasminogen activator; *BAX*: BCL2-Associated X; *NLRP3*: NOD-like receptor thermal protein domain associated protein 3; Chr: Pos: Chromosome and position; eQTL: expression quantitative trait locus. Pathway*: For pathway, we selected the one with the most significant *P*-value in the database. Data comes from eQTLGen - cis-eQTLs

The rs10754558 polymorphism of the *NLRP3* gene may be associated with an increased risk of COM in this Chinese Han population; to the best of our knowledge, no study has reported on this association to date. Although we did not find a difference in the frequency of the mutant alleles, C and G, of rs10754558 in the patient group, the heterozygous model of rs10754558 showed a statistical association of this polymorphism with COM, indicating that individuals with the CG genotype of this polymorphism are in an increased risk of COM development. Other genotypes did not significantly associated with COM. In addition, although there was no statisticalcorrelation between the results of the recessive and homozygous models of rs7525979 (*P* = 0.093 and 0.073, respectively), the frequency of the TT genotype was higher in the COM patients than that in healthy controls. Further research is required to verify whether patients with TT genotype of rs7525979 are in a lower risk to develop thisdisease. Several possible reasons may help explain why only the rs10754558 variants showed significant results while the others failed. The first reason may rest with the specificity of rs10754558 itself. Aside from the present findings, we also found that this SNP is associated with the development of several disorders, such as chronic kidney disease [40], acne vulgaris [41] and autoimmune diseases [42]. Second, the limited sample size of our study may have masked the correlations between the other SNPs and COM susceptibilities. Therefore, we will explore whether and how these genetic variants play roles in epigenetics in the future. Moreover, no significant differences were found among different genotypes of rs10754558 or rs7525978 SNPs, neither regarding clinical characteristics, nor regarding serological levels of IL-6 or TNF-α. These results suggest that further studies are required to investigate the potential influences of *NLRP3* polymorphisms on clinical features and serum levels of the inflammatory biomarkers as well. Nonetheless, outcomes of the present study also need to be confirmed by a larger cohort.

Our study had several limitations. Firstly, despite a larger sample size of the current report compared to the majority of the previously published studies, it remains inadequate for an SNP analysis. Additionally, a broader pool of participants should be applied in future research since the pathogenic process for the development of COM is multifactorial. Secondly, a few participants could not be genotyped using the SNapShot method. Owing to the limited volume of blood collection from each participant, genotyping could not be repeated using alternative methods, resulting in a reduced number of samples with sequencing outcomes. However, this did not affect the final results. Thirdly, we did not detect serum level of *IL-1β*, thus, *IL-1β* levels among patients with different genotypes could not be compared, and correlations between the *NLRP3* gene polymorphisms and serum *IL-1β* levels remain unclear. Fourthly, the environmental variables may play a role in the pathophysiology of COM in addition to the genetic factors. The majority of the patients in this study were transferred from neighboring hospitals; thus, it was difficult to determine their precise medical history prior to bone infection (e.g., the severity of the original injury and treatment approach). As a result, we were unable to further investigate how these external factors affected the development of COM. Finally, we found that *NLRP3* gene polymorphisms are associated with an increased risk of COM; however, the function of the genetic variants has not been analyzed. Therefore, further functional validations of *NLRP3* gene polymorphisms are needed.

## Conclusions

This study confirmed that *NLRP3* gene rs10754558 polymorphism is significantly associated with the prevalence of COM in a Chinese Han population. Nonetheless, this conclusion requires validation by future studies with a larger sample size. Based on our findings, detecting overexpression of the *NLRP3* mutant gene may be a promising strategy to identify risk group for COM development. Future studies exploring this association that include a larger population of COM patients are essential, especially if genetic screening is to be employed as a prediction tool for the risk of disease development. Further, determining whether pharmacological inhibition of the *NLRP3* pathway should be incorporated into the treatment regimen for COM may be beneficial.

### Electronic supplementary material

Below is the link to the electronic supplementary material.

Supplementary Material 1

Supplementary Material 2

## Data Availability

The datasets generated and analyzed during the current study are not publicly available to respect and protect the privacy of the patients; however, they are available from the corresponding author upon reasonable request.

## Abbreviations

NLRP3: NOD-like receptor thermal protein domain associated protein 3
COM: Chronic osteomyelitis
OM: Osteomyelitis
OR: Odds ratio
CI: Confidence interval
IQR: Interquartile range
IL: Interleukin
SNPs: Single nucleotide polymorphisms
TNF-α: Tumor necrosis factor-α
HWE: Hardy-Weinberg equilibrium
PTOM: Post-traumatic osteomyelitis
VDR: Vitamin D receptor
tPA: Tissue plasminogen activator
COX-2: Cyclooxygenase-2
MMP-1: Matrix metalloproteinase-1
